# Superparamagnetic Nanocrystals Clustered Using Poly(ethylene glycol)-Crosslinked Amphiphilic Copolymers for the Diagnosis of Liver Cancer

**DOI:** 10.3390/pharmaceutics15092205

**Published:** 2023-08-25

**Authors:** Ling Jiang, Jiaying Chi, Jiahui Wang, Shaobin Fang, Tingting Peng, Guilan Quan, Daojun Liu, Zhongjie Huang, Chao Lu

**Affiliations:** 1 Department of Pharmacy, Shantou University Medical College, Shantou 515041, China; 2 College of Pharmacy, Jinan University, Guangzhou 511436, China; 3 The Second Affiliated Hospital of Shantou University Medical College, Shantou 515000, China; 4 Department of Radiology, Shenzhen Longhua Maternity and Child Healthcare Hospital, Shenzhen 518109, China

**Keywords:** amphiphilic copolymer, superparamagnetic iron oxide, shell-crosslinked micelle, contrast agent, magnetic resonance imaging

## Abstract

Superparamagnetic iron oxide (SPIO) nanocrystals have been extensively studied as theranostic nanoparticles to increase transverse (T_2_) relaxivity and enhance contrast in magnetic resonance imaging (MRI). To improve the blood circulation time and enhance the diagnostic sensitivity of MRI contrast agents, we developed an amphiphilic copolymer, PCPZL, to effectively encapsulate SPIO nanocrystals. PCPZL was synthesized by crosslinking a polyethylene glycol (PEG)-based homobifunctional linker with a hydrophobic star-like poly(ε-benzyloxycarbonyl-L-lysine) segment. Consequently, it could self-assemble into shell-crosslinked micelles with enhanced colloidal stability in bloodstream circulation. Notably, PCPZL could effectively load SPIO nanocrystals with a high loading capacity of 66.0 ± 0.9%, forming SPIO nanoclusters with a diameter of approximately 100 nm, a high cluster density, and an impressive T_2_ relaxivity value 5.5 times higher than that of Resovist^®^. In vivo MRI measurements highlighted the rapid accumulation and contrast effects of SPIO-loaded PCPZL micelles in the livers of both healthy mice and nude mice with an orthotopic hepatocellular carcinoma tumor model. Moreover, the magnetic micelles remarkably enhanced the relative MRI signal difference between the tumor and normal liver tissues. Overall, our findings demonstrate that PCPZL significantly improves the stability and magnetic properties of SPIO nanocrystals, making SPIO-loaded PCPZL micelles promising MRI contrast agents for diagnosing liver diseases and cancers.

## 1. Introduction

Magnetic resonance imaging (MRI) is a noninvasive and nonradiative imaging technique used for molecular analysis and medical diagnosis [1,2,3,4]. It utilizes the differences in magnetic susceptibility values between tissues, such as tumors and normal tissues, to generate contrast. Several contrast agents have recently been developed to enhance the diagnostic accuracy and sensitivity of MRI. For example, superparamagnetic iron oxide (SPIO) nanocrystals are considered effective T_2_-weighted imaging contrast agents that produce darker images against a bright background of water or tissue by causing protons in their vicinity to undergo spin–spin relaxation [5,6,7,8]. However, common preparation methods frequently produce SPIO nanocrystals with a hydrophobic surfactant layer on their surface, which reduces their dispersion in water and ultimately leads to rapid clearance from the body and low sensitivity of MRI [9,10].

To enhance the quality of MRI, researchers have proposed the use of amphiphilic polymers to encapsulate hydrophobic SPIO nanocrystals and facilitate their dispersion in aqueous solutions through micelle formation [2,11,12]. Moreover, several studies have shown that these strategies have significant potential for improving the sensitivity of MRI [13,14,15]. A significant enhancement in T_2_ relaxivity (r_2_) can be achieved using synergistic magnetism through the encapsulation of multiple SPIO nanocrystals in the hydrophobic core of micelles to form magnetic nanoclusters [16,17]. The aggregation state, including cluster density and size, plays an important role in regulating the physical interactions between the SPIO nanocrystals and their magnetization. While the r_2_ value increases with a decrease in the interparticle spacing of the SPIO nanocrystals, a higher r_2_ value is typically observed with an increase in the diameter of the SPIO nanoclusters. Both cluster density and size can be tailored by adjusting the polymer-to-SPIO ratio of the SPIO-loaded micelles [18,19]. The findings of previous studies suggest that the MRI effect of SPIO-loaded micelles is highly dependent on the performance of the carriers, which is necessary to achieve high drug-loading capacity and stability.

**Scheme 1:**
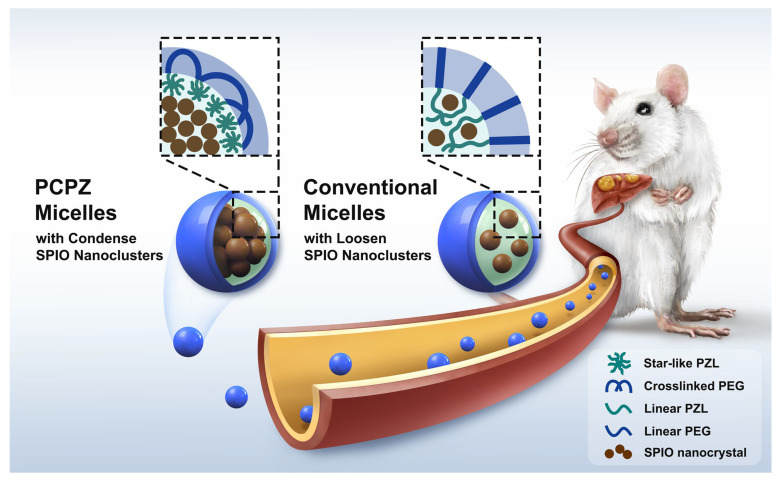
Schematic illustration of the structural differences between SPIO-loaded micelles based on the PCPZL and conventional linear amphiphilic copolymers to diagnose liver disease or cancer.

Inspired by these findings, we used a polyethylene glycol (PEG)-based homobifunctional linker to crosslink the star-like hydrophobic block of poly(ε-benzyloxycarbonyl-L-lysine) (PZL). Subsequently, we obtained a series of amphiphilic PEG-crosslinked PZL (PCPZL) for SPIO nanocrystal loading. This polymer represented a promising copolymer with molecular weights several orders of magnitude higher than those of small-molecular amphiphiles, as well as potential carriers for forming polymeric micelles with enhanced stability and drug loading capacity [20,21,22]. PCPZL is expected to be a high-performance SPIO nanocrystal carrier for the formation of magnetic nanoclusters, thereby enhancing T_2_ relaxivity in MRI and facilitating the diagnostic sensitivity of liver diseases or cancers, as described below (Scheme 1): (1) the PEG-crosslinked amphiphiles may self-assemble into polymeric micelles with a crosslinked PEG shell in an aqueous solution, ensuring improved water solubility, colloidal stability, and long-term circulation via the “stealth effect” [23]. (2) the amphiphilic structure of PCPZL may endow the carrier with not only enhanced stability, but also a high drug-loading capacity [20,21,22], which may increase the r_2_ value by facilitating the formation of SPIO nanoclusters and increasing the cluster size in the micelle core. (3) The star-like PZL hydrophobic block with multiple but short side chains may occupy less interparticle spacing of the SPIO nanocrystals than other linear but long hydrophobic blocks, which is beneficial for increasing the SPIO cluster density and consequently further enhancing the r_2_ value.

## 2. Materials and Methods

### 2.1. Materials

Hyperbranched polyethylenimine (PEI, M_W_ = 0.8 kDa) and bis(trichloromethyl) carbonate (triphosgene) were obtained from Sigma-Aldrich (St. Louis, MO, USA). ε-benzyloxycarbonyl-L-lysine was supplied by GL Biochem (Shanghai, China) and used without further purification. PEG (succinimidyl carboxymethyl ester)_2_ (SCM-PEG-SCM, 7.5 kDa) was purchased from Beijing Jenkem Technology Co., Ltd. (Beijing, China). Oleic acid-coated SPIO nanocrystals (1 mg mL^−1^) prepared using co-precipitation were obtained from Nanjing Nanoeast Biotechnology Co., Ltd. (Nanjing, China). A dialysis tube (molecular weight cut-off (MWCO) of 14 kDa) was obtained from Viskase (Darien, IL, USA). All other reagents were of analytical grade and were used as received. Analytically pure dimethyl sulfoxide (DMSO), dichloromethane (DCM), ethyl acetate, acetone, and tetrahydrofuran (THF) were provided by Shantou Xilong Chemical Co., Ltd. (Shantou, China).

### 2.2. Synthesis of Star-like Hydrophobic Copolymers PZL

PZL copolymers with a PEI core and poly(ε-benzyloxycarbonyl-L-lysine) side chains were synthesized using ring-opening polymerization (ROP) of α-amino acid N-carboxyanhydride (NCA), as described in our previous study [24,25]. Briefly, ε-benzyloxycarbonyl-L-lysine N-carboxyanhydride (Lys(Z)-NCA) was prepared via the Fuchs–Farthing method using ε-benzyloxycarbonyl-L-lysine and triphosgene in anhydrous ethyl acetate [26]. Subsequently, Lys(Z)-NCA was dissolved in an anhydrous mixture of DMSO and DCM (1:5 volume ratio). An appropriate amount of PEI initiator solution in DMSO was then added, and the reaction was conducted at room temperature for 36 h. After removal of the DCM solvent via rotary evaporation, the sample was subjected to dialysis against ultrapure water (MWCO of 14 kDa) for four days followed by lyophilization to obtain the desired product.

### 2.3. Synthesis of PEG-Crosslinked Amphiphilic Copolymers PCPZL

PZL (100 mg) was added to 300 mL of an anhydrous mixture of DMSO and DCM (1:5 volume ratio) and stirred until it was fully dissolved. Subsequently, a solution of SCM-PEG-SCM with a concentration of 0.2 g mL^−1^ was prepared by dissolving it in DCM and then adding it dropwise at a rate of 100 μL min^−1^ into the PZL solution under vigorous stirring conditions. After reacting for 36 h at 25 °C, the DCM was removed through rotatory evaporation. The solution was then dialyzed against ultrapure water for four days using a dialysis bag with an MWCO of 14 kDa, and the final products were obtained using lyophilization.

### 2.4. Characterization of Various Copolymers

Proton nuclear magnetic resonance (^1^H NMR) spectra of PZL and PCPZL were collected at room temperature using a Bruker DMX 400 MHz spectrometer (Bruker, Karlsruhe, Germany) using deuterated dimethyl sulfoxide (DMSO-*d*_6_) as the solvent. The molecular weight parameters of the polymers were analyzed using a gel permeation chromatography (GPC) system from Waters (Milford, CT, USA), which consisted of a Waters 515 HPLC pump and a Waters 2414 refractive index detector. The samples were analyzed using dimethylformamide (DMF) as the eluent and linear poly(methyl methacrylate) (PMMA) as the calibration standard.

### 2.5. Fabrication of SPIO-Loaded PCPZL Micelles

The SPIO-loaded magnetic micelles were prepared using an optimized ultrasonic dialysis method. Briefly, oleic acid-coated SPIO nanocrystals containing 15 mg of iron and 10 mg of PCPZL copolymers were co-dissolved in 0.2 mL of THF solvent through continuous vortexing. Subsequently, 1.8 mL of acetone solution was added dropwise, and the mixture was shaken for 1 h to completely dissolve the SPIO nanocrystals through the hydrotropic action facilitated by PCPZL. This solution was slowly added dropwise (at a rate of one drop every 10 s) to 4 mL of ultrapure water and subjected to sonication (40 kHz, 100 W). Sonication was continued for an additional 30 min after the complete addition of the solution, followed by dialysis against water for 24 h to remove organic solvents, including THF and acetone (MWCO = 14 kDa). Finally, the free SPIO nanoparticles that were not loaded into the micelles were removed through two rounds of centrifugation at 3000 rpm for 10 min, and the resulting SPIO-loaded PCPZL magnetic micelles were then stored at 4 °C for further investigation.

### 2.6. Characterization of SPIO-Loaded PCPZL Micelles

#### 2.6.1. Loading Capacity and Loading Efficiency Characterizations

The loading capacity and loading efficiency of SPIO nanocrystals in the magnetic micelles were determined by quantifying the Fe content using the phenanthroline spectrophotometric method [27]. Briefly, 50 μL of SPIO-loaded PCPZL micelle solution was treated with two drops of concentrated HCL solution for 12 h to completely degrade the SPIO-loaded micelles. Subsequently, 2.5 mL of acetic acid–sodium acetate buffer solution was added and vortexed for 3 h, followed by the addition of another 1.25 mL of 1% (*w*/*v*) hydroxylammonium chloride solution and 2.5 mL of 0.1% (*w*/*v*) 1,10-phenanthroline solution. The solution was then diluted and mixed with ultrapure water in a volumetric flask to obtain a final volume of 25 mL. After incubation for 10 min, the absorbance at a wavelength of 510 nm was measured, and the SPIO concentration was determined based on a previously established Fe^2+^ calibration curve.

The SPIO loading capacity was calculated using the following equation:

(1) $$\mathrm{Loading}\mathrm{capacity}\left(\%\right)=\frac{\mathrm{Weight}\mathrm{of}\mathrm{encapsulated}\mathrm{iron}\mathrm{oxide}}{\mathrm{Weight}\mathrm{of}\mathrm{encapsulated}\mathrm{iron}\mathrm{oxide}+\mathrm{Weight}\mathrm{of}\mathrm{initial}\mathrm{PCPZL}}\times 100\%$$

The SPIO loading efficiency was calculated using the following equation:

(2) $$\mathrm{Loading}\mathrm{efficiency}\left(\%\right)=\frac{\mathrm{Weight}\mathrm{of}\mathrm{encapsulated}\mathrm{iron}\mathrm{oxide}}{\mathrm{Weight}\mathrm{of}\mathrm{initial}\mathrm{iron}\mathrm{oxide}}\times 100\%$$

#### 2.6.2. Particle Sizes and Zeta Potential Characterizations

The particle sizes and zeta potential of the SPIO-loaded PCPZL micelles were recorded using a Zetasizer Nano ZS90 (Malvern Instruments, Worcestershire, UK). The micelle solution was appropriately diluted with ultrapure water and introduced into the sample pool to measure its diameter at a scattering angle of 90° at room temperature using a refractive index of 1.590 and a medium viscosity of 0.887 cP. For the zeta potential measurement, the sample was diluted with phosphate buffer (PBS) at pH 7.4 and subsequently determined using the same instrument in a disposable folded capillary zeta cell at room temperature.

#### 2.6.3. Morphological Studies

Transmission electron microscopy (TEM) was conducted at room temperature using a JEM 2100F electron microscope operated at 200 kV. The samples were prepared by drying a dispersion of nanoparticles on a copper grid coated with amorphous carbon. The diameter distribution histograms of the SPIO nanocrystals and SPIO nanoclusters were fitted to a peak model and visualized using Origin 9.1 (Origin Lab, Northampton, MA, USA).

#### 2.6.4. Critical Micelle Concentration (CMC) Measurement

The CMC of PCPZL was determined using fluorescence, employing pyrene as the probe molecule. Samples of micellar solution with concentrations ranging from 1 × 10^−5^ to 1 × 10^−1^ mg mL^−1^ were prepared and then left to equilibrate with a constant pyrene concentration of 6 × 10^−7^ mol L^−1^ for 24 h. The excitation intensity of pyrene at 339 and 334 nm was obtained using a fluorescence spectrometer (PTI QM-TM, Horiba Jobin Yvon Inc., Edison, NJ, USA) according to the scanning excitation spectrum between 300 and 360 nm by fixing the emission spectrum at 390 nm. The CMC value was determined from the intersection points of the tangents corresponding to lower and higher polymer concentrations.

### 2.7. Magnetization Measurement

Lyophilized samples of both SPIO nanocrystals and SPIO-loaded magnetic micelles were weighed, and the magnetization data were determined using an MPMS XL-7 Quantum Design SQUID magnetometer at room temperature. The applied magnetic field was varied from 2 × 10^4^ Oe to −2 × 10^4^ Oe to generate hysteresis loops.

### 2.8. T_2_ Relaxivity Measurement

A Signa HDxt 1.5 T MRI scanner (GE Medical Systems, Milwaukee, WI, USA) was used to measure the relaxivity of the SPIO magnetic micelle solution after the appropriate dilution with ultrapure water at various Fe concentrations ranging from 0 to 20 μg mL^−1^. For the T_2_-weighted fast spin-echo (FSE) sequence, the parameters were set as follows: repetition time (TR) = 1000 ms and echo time (TE) = 8.6, 17.2, 25.8, 34.4, 43.0, 51.6, 60.3, and 68.9 ms. The r_2_ value was determined from the slope of the transverse relaxation rate (1/T_2_) versus the Fe concentration of the SPIO-loaded micelles. 

### 2.9. In Vitro MRI

An in vitro MRI experiment was performed using a Trio Tim 3.0 T MRI instrument (Siemens AG SI, Erlangen, Germany) at room temperature. T_2_-weighted images were acquired using the turbo-spin echo (TSE) sequence with the following parameters: head surface coil, axial scanning, TR = 5000 ms, and TE = 93 ms. The solutions of SPIO-loaded PCPZL micelles containing different Fe concentrations (0, 0.625, 1.25, 2.5, 5, 10, and 20 μg mL^−1^) were prepared in ultrapure water and then placed in 1.5 mL centrifuge tubes for MRI scanning.

### 2.10. In Vivo Mouse Liver and Liver Tumor Imaging

All animal procedures were performed according to the Guidelines for Care and Use of Laboratory Animals of Shantou University Medical College, and the experiments were approved by the Animal Ethics Committee of Shantou University Medical College (SUMC2016-200). For in vivo mouse liver imaging, healthy Kunming mice weighing 21–25 g were anesthetized using intraperitoneal (i.p.) injection of pentobarbital sodium and then intravenously injected with the SPIO contrast agent at a dose of 5 mg Fe/kg body weight via the tail vein. MRI scanning was performed before and 3, 15, 30, and 60 min after the administration of the magnetic micelles. T_2_-weighted TSE images were acquired using a Trio Tim 3.0 T MRI instrument (Siemens AG SI, Germany) with the following scanning parameters: TR/TE = 4000/92 ms and slice thickness = 3 mm. After scanning, the images were transferred to an ADW4.4 workstation (GE Healthcare) to measure the MRI signal intensities in the regions of interest.

For in vivo mouse liver tumor imaging, BALB/c nude mice weighing 20 ± 2 g were used to establish a tumor model of orthotopic hepatocellular carcinoma [28,29,30]. The mice were provided food and water ad libitum and acclimatized for 1 week before the start of the experiment. After anesthesia and disinfection, a 1 cm incision was made in the abdomen to expose the liver. Subsequently, a 0.03 mL suspension of human liver cancer cells SMMC-7721 (1 × 10^8^ cells per mL) was injected into the liver before it was sutured and allowed to grow for 15 days. Subsequently, nude mice bearing human hepatocellular carcinoma were anesthetized via i.p. injection of 1% pentobarbital sodium, and solutions of the SPIO-loaded PCPZL micelles were injected into the nude mice via the tail vein at a dose of 5 mg Fe/kg body weight. The animals were then scanned using a 3.0 T MR scanner (Siemens Prisma, Erlangen, Germany) with a gradient strength up to 80 mT/m. T_2_-weighted images were then acquired using a FSE sequence (TR = 6000 ms, TE = 20 ms). The signal intensity values in the defined regions of interest were collected at 0, 5, 10, 30, 60, 120, 180, 480, 720, 960, and 1200 min.

## 3. Results and Discussion

### 3.1. Synthesis and Characterization of PEG-Crosslinked Amphiphilic Copolymers PCPZL

**Figure 1:**
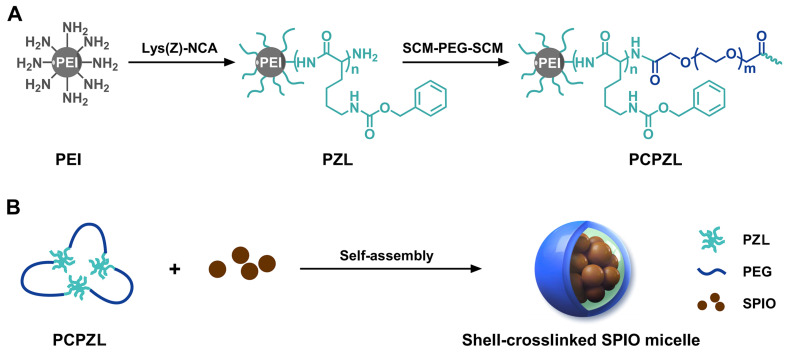
Schematic of the preparation of SPIO-loaded PCPZL micelles. (**A**) Synthesis route of PCPZL copolymer. (**B**) Schematic representation of shell-crosslinked magnetic micelles self-assembled from PCPZL copolymers and hydrophobic SPIO nanocrystals.

In this study, a PEG-crosslinked amphiphilic copolymer, PCPZL, was synthesized in two steps (Figure 1) following an established and optimal protocol reported in our previous research [22,24,25]. First, the star-like hydrophobic copolymers, PZL, were constructed by grafting PEI with hydrophobic poly(ε-benzyloxycarbonyl-L-lysine) using a one-step ring-opening polymerization method [25]. The polymerization of Lys(Z)-NCA was initiated by the terminal amino groups of PEI, which had a molecular weight of 0.8 kDa and approximately eight peripheral primary amines [31]. The degree of polymerization and length of each poly(ε-benzyloxycarbonyl-L-lysine) side chain were easily controlled by adjusting the feeding ratio of the Lys(Z)-NCA monomer to PEI. Therefore, we synthesized two types of star-like copolymers, PZL1 and PZL2, which had a PEI (M_W_ = 0.8 kDa) core and eight poly(ε-benzyloxycarbonyl-L-lysine) chains with degrees of polymerization of three and six, respectively.

**Figure 2:**
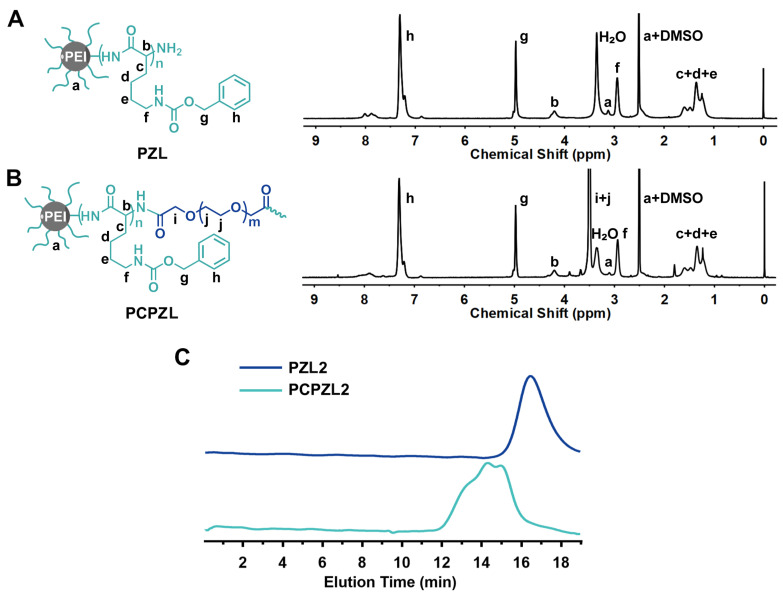
Structural characterization of various copolymers. ^1^H NMR spectrum of PZL2 (**A**) and PCPZL2 (**B**) in DMSO-*d*_6._ (**C**) GPC chromatograms of various copolymers.

Then, the homobifunctional linker SCM-PEG-SCM was employed to crosslink PZL and obtain the amphiphilic copolymer PCPZL [22,24]. To achieve a mass ratio of approximately 1:1 between the hydrophilic block PEG and hydrophobic block PZL, we synthesized PCPZL at feeding molar ratios of SCM-PEG-SCM to PZL1 and PZL2 of 1:1 (PCPZL1) and 2:1 (PCPZL2), respectively. We calculated the actual molar ratio of PEG and the PZL segment in the product by analyzing ^1^H NMR spectra of various polymers utilizing the integration peaks at 3.4–3.7 ppm (corresponding to the methylene groups of the PEG block, OCH_2_CH_2_O-) compared with the peaks at 5.0 ppm (corresponding to the methylene groups of the poly(ε-benzyloxycarbonyl-l-lysine) block, -OCH_2_-C_6_H_5_) (Figure 2A,B and Table 1). As shown in Table 1, the measured molar ratio of PEG to the PZL segment in both PCPZL1 and PCPZL2 closely approximated the feeding molar ratio of SCM-PEG-SCM to PZL. Moreover, the mass ratio of PEG to the PZL segment in both copolymers was approximately 1:1, which was considered to achieve an optimal hydrophilic–hydrophobic balance for amphiphilic polymers [22].

The molecular weights and molecular weight distributions were determined using GPC measurements. As shown in Figure 2 and Table 1, the number average molecular weights of PCPZL1 and PCPZL2 were calculated to be 12.57 and 26.99 kDa, respectively, which are significantly higher than those of PZL1 and PZL2 (4.23 and 6.52 kDa). In addition, all the samples exhibited a narrow molecular weight distribution with a polymer dispersity index (PDI) below 1.50. The GPC results demonstrated a successful crosslinking reaction induced by SCM-PEG-SCM, and the obtained PCPZL1 and PCPZL2 possessed appropriate molecular weights for drug delivery applications.

### 3.2. Preparation and Characterization of SPIO-Loaded PCPZL Micelles

The amphiphiles obtained after PEG crosslinking have shown superior ability to effectively encapsulate hydrophobic drugs and form polymeric micelles with enhanced stability and drug loading capacity in our previous studies [22]. This study further explores the potential of such systems in facilitating the formation of magnetic nanoclusters, thus enhancing T_2_ relaxivity in MRI and facilitating the diagnostic sensitivity of liver diseases or cancers. SPIO-loaded PCPZL micelles were fabricated using an optimized ultrasonic dialysis method. The PCPZL copolymer and SPIO nanocrystals were uniformly dispersed in the organic solution. Subsequently, the self-assembly of SPIO-loaded micelles occurred spontaneously through the mixing of the organic and aqueous phase solutions. The hydrophobic environment of hydrophobic SPIO nanocrystals spontaneously distributed in the core of the micelles was driven by hydrophobic effects, whereas the crosslinked PEG chains distributed in the outer layer of the micelles primarily played the role of forming a stable hydration outer layer to protect the micelles [32,33,34]. Ultrasound played a crucial role in the preparation of SPIO-loaded PCPZL micelles by regulating the size distribution of the micelles and facilitating the conformational adjustment of SPIO nanoclusters within the micelles to increase their density.

**Figure 3:**
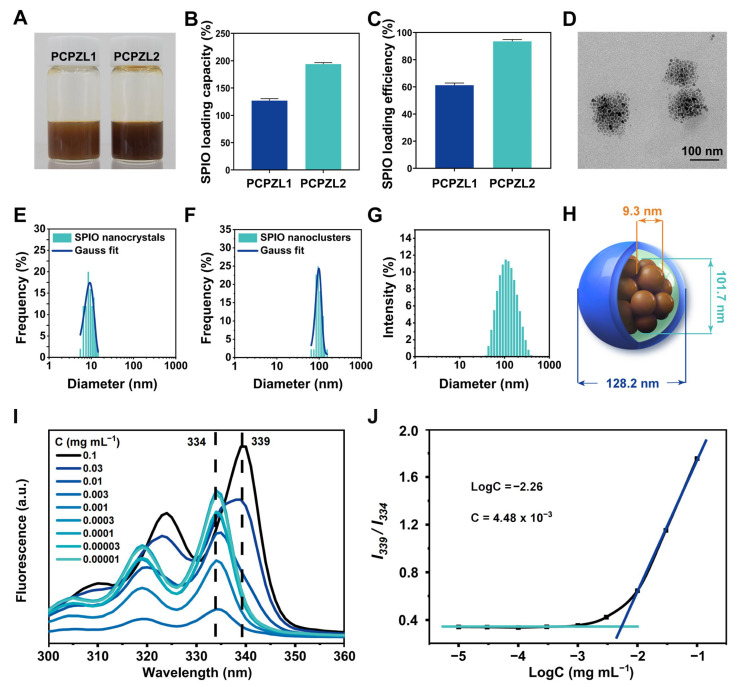
Structural characterization of SPIO-loaded micelles. (**A**) Photograph of various SPIO-loaded micelle solutions. (**B**) SPIO loading capacity and (**C**) SPIO loading efficiency of various micelles. (**D**) TEM image of SPIO-loaded PCPZL2 micelles. Size distribution of (**E**) SPIO nanocrystals and (**F**) SPIO nanoclusters inside the SPIO-loaded PCPZL2 micelles with Gauss fitting. (**G**) Hydrodynamic diameter of SPIO-loaded PCPZL2 micelles determined using DLS. (**H**) Schematic of the structural composition of SPIO-loaded PCPZL2 micelles. (**I**) Fluorescence spectrum of pyrene in PCPZL2 aqueous solutions with a constant pyrene concentration (6 × 10^−7^ mol L^−1^) and various concentrations of PCPZL2. (**J**) Plot of *I*_339_/*I*_334_ of pyrene versus logarithm concentration of PCPZL2.

The prepared SPIO-loaded PCPZL1 and PCPZL2 micelles are shown in Figure 3A. The PCPZL2 magnetic micelles were darker than that of the PCPZL2 magnetic micelles, indicating that PCPZL2 was more capable of loading SPIO nanocrystals. The results of quantitative detection confirmed that the SPIO loading capacities of the PCPZL1 and PCPZL2 micelles were 55.9 ± 1.6% and 66.0 ± 0.9%, respectively, and the corresponding SPIO loading efficiencies were measured at 61.2 ± 1.6% and 93.5 ± 1.3% (Figure 3B,C). The high SPIO loading capacity of PCPZL2 micelles is significantly higher compared with those reported in the relevant literature [19,35,36]. It has been reported that a high SPIO loading capacity of micelles (i.e., an increase in the SPIO/polymer mass ratio) increases the diameter of the SPIO nanoclusters, with a corresponding gradual increase in T_2_ relaxivity [19]. 

Subsequently, we employed TEM and dynamic light scattering (DLS) measurements to observe the SPIO distribution in the polymeric micelles and determine the diameter of the SPIO-loaded PCPZL2 micelles, respectively (Figure 3D–H). Figure 3d confirms that the SPIO nanocrystals were clustered in the core of the PCPZL2 micelles with a high cluster density. An analysis of the TEM image revealed that SPIO nanocrystals, with an average diameter of 9.3 ± 2.1 nm, were encapsulated within the micelles and formed approximately 101.7 ± 17.2 nm SPIO nanoclusters (Figure 3E,F). The DLS analysis showed that although the SPIO nanoclusters of SPIO-loaded PCPZL2 micelles had a large diameter; the average hydrodynamic diameter of this magnetic micelle was only 128.2 nm (Figure 3G). This may be attributed to the low polymer content in the micelles and the crosslinked structure of PEG, which limited the extension of the PEG chain segments on the nanoparticle surface and the size of the hydration layer (Scheme 1). Moreover, studies have reported that the hydrodynamic size of nanoparticles plays a significant role in their plasma half-life, biological distribution, and pharmacokinetics [37]. Nanoparticles with a diameter of approximately 100 nm may exhibit a prolonged circulation time in vivo, which facilitates the targeted accumulation of contrast agents at specific organs or sites and ultimately enhances the quality of MRI [38,39].

In addition, we conducted a zeta potential analysis on SPIO-loaded PCPZL2 micelles and found that the micelles had a zeta potential of +0.03 mV. The approximate neutral zeta-potential is consistent with the hypothesis that the outer shell of these micelles is composed primarily of PEG rather than PEI. This may also be attributed to the fact that PEI only occupies 5.9% of the mass ratio of the PZL2 segment. The colloidal stability of the delivery system primarily describes whether the system will aggregate and precipitate during long-term storage. Therefore, SPIO-loaded PCPZL2 micellar suspensions were stored at 4 °C for 1 month and were then examined for their particle size distribution. The average hydrodynamic diameter of the micelles was determined to be 133.5 nm, showing slight changes during storage, while no visible precipitation was observed. The results indicated that the colloidal stability of the SPIO-loaded PCPZL2 micelles was largely high, which may be attributed to the fact that the crosslinked PEG shell provides a stable hydration layer to inhibit the aggregation of nanoparticles.

The main challenge faced by micelles in the bloodstream is their susceptibility to disaggregation upon significant blood dilution, resulting in premature drug leakage. The critical micelle concentration (CMC) is commonly employed to evaluate the thermodynamic stability of micelles upon dilution [40]. In this study, pyrene was used as a hydrophobic fluorescent probe to determine the CMC of PCPZL2. The typical excitation fluorescence spectra of pyrene in PCPZL2 aqueous solutions with a constant pyrene concentration (6 × 10^−7^ mol L^−1^) and various concentrations of PCPZL2 are shown in Figure 3I. By plotting the logC against the ratio of fluorescence intensity at 339 nm and 334 nm (*I*_339_/*I*_334_), two straight lines with different slopes were obtained, and the CMC value was thus determined from the inflection point on the plot. As shown in Figure 3J, the CMC of PCPZL2 was determined to be 4.48 × 10^−3^ mg mL^−1^, which was 10 to 100 times lower than that of many conventional linear polymers [34]. The results indicated an adequate stability of the shell-crosslinked PCPZL2 micelles upon dilution, for example, in the bloodstream.

### 3.3. Magnetization and T_2_ Relaxivity of SPIO-Loaded Micelles

**Figure 4:**
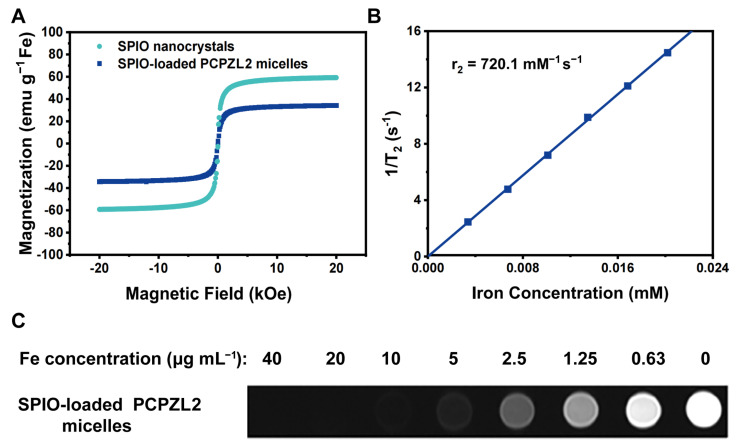
Magnetization and T_2_ relaxivity measurement of SPIO-loaded micelles. (**A**) Hysteresis loops of the SPIO nanocrystals and SPIO-loaded PCPZL2 micelles. (**B**) T_2_ relaxation rate (1/T_2_) as a function of the iron concentration (mM) for SPIO-loaded PCPZL2 micelles, and the slope indicates the specific r_2_. (**C**) T_2_-weighted MRI images of SPIO-loaded PCPZL2 micelles at different iron concentrations.

The magnetic properties of SPIO-loaded PCPZL2 micelles were determined using a vibrating sample magnetometer [41]. Because the magnetization of superparamagnetic materials did not linearly depend on the applied field, the shape of this hysteresis line indicated that the SPIO-loaded PCPZL2 micelles were superparamagnetic (Figure 4A). The saturation magnetization values of single SPIO nanocrystals and SPIO-loaded PCPZL2 micelles reached maximum values of 59.1 and 34.2 emu g^−1^ Fe, respectively. The slight saturation magnetization reduction of the SPIO-loaded PCPZL2 micelles may be attributed to the encapsulation of the polymer; a similar phenomenon has also been observed in other polymer-coated magnetic nanocluster systems [42,43]. Because a saturation magnetization of 9.7–30.3 emu g^−1^ Fe has been reported to be sufficient for biomedical applications of T_2_ contrast agents [44], SPIO-loaded PCPZL2 micelles with saturation magnetization of 34.2 emu g^−1^ Fe can be used for T_2_-weighted MRI.

To evaluate the performance of the magnetic nanocrystal-loaded micelles as MRI contrast agents, we studied their relaxivity using a 1.5 T MRI scanner. T_2_ contrast agents can shorten T_2_ (spin–spin) relaxation times, and their net effectiveness is frequently denoted as r_2_, which represents the reciprocal of the relaxation time per unit of iron concentration [45,46]. Studies have reported the r_2_ values of commercialized SPIO nanoparticles Resovist^®^ and Feridex^®^ as 130 and 108 mM^−1^ s^−1^, respectively [9,47]. However, our finding indicated that the r_2_ value of SPIO-loaded PCPZL2 micelles reached 720.1 mM^−1^ s^−1^ (Figure 4B), which was approximately 5.5 and 6.7 times higher than that of Resovist^®^ and Feridex^®^, respectively. The high r_2_ value of the SPIO-loaded PCPZL2 micelles was attributed to the high SPIO loading capacity as well as the diameter and density of the SPIO nanoclusters. 

To evaluate the potential of SPIO-loaded PCPZL2 micelles as MRI contrast agents, we used an MRI scanner to obtain T_2_-weighted images of the micelles at different concentrations at room temperature. Figure 4C shows that as the concentration of the solution increased, the T_2_-weighted imaging signal of the agents gradually decreased and changed from light to dark. As evidenced by the complete darkening of the MRI images at iron concentrations as low as 10 μg·mL^−1^, the SPIO-loaded PCPZL2 micellar solution can be considered as a promising MRI contrast agent. The amphiphilic copolymer PCPZL also has significant potential for clustering SPIO-based T_2_ agents and forming magnetic micelles, thereby enhancing their sensitivity in T_2_-weighted MRI.

### 3.4. In Vivo Mouse Liver MRI Study

**Figure 5:**
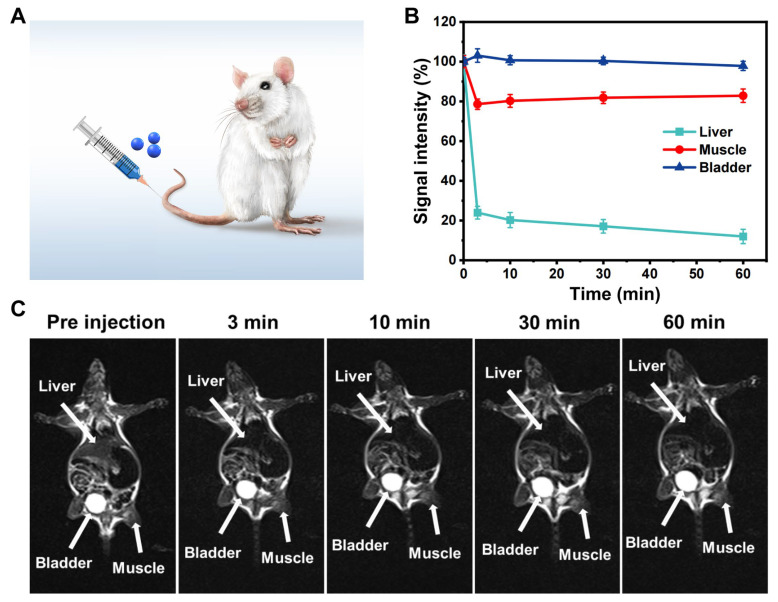
In vivo T_2_-weighted MRI performed on the liver of mice. (**A**) Schematic of mice intravenously injected with SPIO-loaded PCPZL2 micelles. (**B**) Time-dependence of the relative signal intensity in the livers, muscles, and bladders of mice before and after tail vein administration of SPIO-loaded PCPZL2 micelles. (**C**) Mouse T_2_-weighted images of mice at different time points before and after administration of SPIO-loaded micelles.

The SPIO-based contrast agents Resovist^®^ and Feridex^®^ have been extensively used in clinical practice for liver-specific MRI [48]. Therefore, this study aimed to evaluate the liver MRI performance of SPIO-loaded PCPZL2 micelles using Kunming mice as model animals. Figure 5 shows representative T_2_-weighted MRI images of mice before and after injection of SPIO-loaded PCPZL2 micelles. Regions of interest were selected for the images of the bladder, muscles, and liver to quantify the contrast enhancement. The bladder area in all the images appeared bright white due to its high water content. The muscles in the thighs of the mouse also did not visibly turn black, which may have been because the SPIO-loaded PCPZL2 micelles did not accumulate at high concentrations in the muscle tissue within 1 h. In contrast, a significant enhancement was observed in the liver after a 3 min injection of SPIO-loaded PCPZL2 magnetic micelles, and quantitative analysis showed an approximately 80% reduction in the signal (Figure 5C). The signal in the liver continued to decrease and maintained a strong enhancement effect 60 min after injection, likely because nanoparticles with diameters of approximately 100 nm easily accumulated through passive targeting in the liver. In addition, the results showed that SPIO-loaded PCPZL2 micelles had a long half-life in the liver, which was beneficial for their distribution and accumulation in specific areas to provide more detailed MRI images.

**Figure 6:**
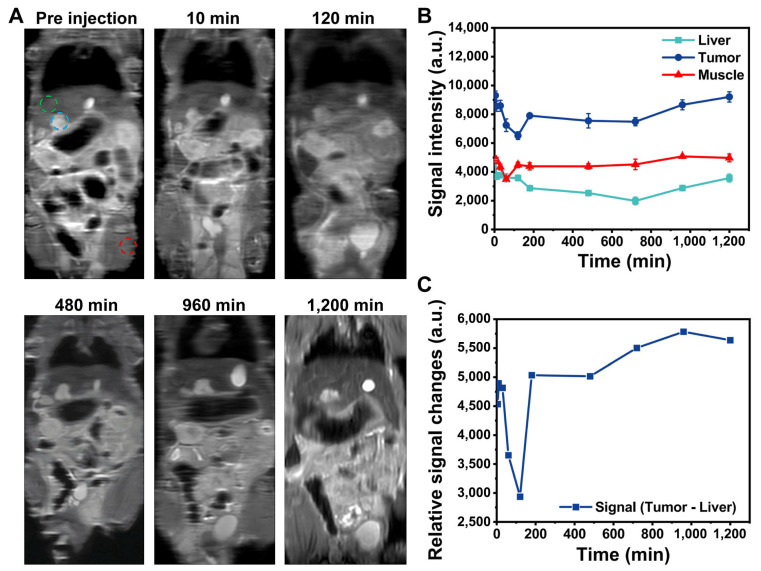
In vivo T_2_-weighted MRI of nude mice bearing human hepatocellular carcinoma. (**A**) T_2_-weighted images of mice at different time points before and after tail vein administration of SPIO-loaded PCPZL2 micelles. The blue, green, and red circles indicate the regions of interest for the tumor, liver, and muscle, respectively, selected for the signal intensity measurement. (**B**) Mean values of signal intensity collected before and after micelle administration. (**C**) Time-dependence of the relative signal difference between tumor and normal liver tissues at different time points.

To evaluate the potential of SPIO-loaded PCPZL2 micelles as MRI contrast agents for tumor imaging, we performed in vivo MRI of nude mice bearing human liver cancer using an MRI scanner in which signals from the liver, muscle, and tumor were measured at the workstation. Figure 6A,B shows that the SPIO-loaded PCPZL2 micelles afforded noticeably shortened MRI T_2_ and a significantly low MRI signal intensity within the tumors and livers of BALB/c nude mice over a period of 1200 min. The prolonged MRI signal observed in these tissues is likely attributed to the “stealth effect” of the crosslinked PEG shell [22], reducing SPIO clearance from the body.

Although SPIO-loaded PCPZL2 micelles may accumulate non-specifically in the liver, there are significant structural (e.g., vascular) and metabolic differences between tumors and normal liver tissues, which can affect the distribution and duration of magnetic nanoparticles in the two different tissues. As a result, the magnetic nanoparticles would exhibit a differential impact on the MRI signal intensity of these two tissues, and an optimal display period needs to be sought during the clinical use of MRI contrast agents. In this study, the MRI signal intensity of the mouse liver reached its lowest value 720 min after injection of SPIO-loaded PCPZL2 micelles and then gradually recovered, whereas the MRI signal intensity of the mouse liver tumor reached its lowest value 120 min after injection. Figure 6C shows that the relative signal difference between the tumor tissue and the normal liver tissue reached a maximum at 960 min. Therefore, this time point was considered to be the optimal time for diagnosis after injecting the SPIO-loaded PCPZL2 micelles, as it enabled easy detection and observation of lesion details.

## 4. Conclusions

In this study, the amphiphilic copolymers PCPZL1 and PCPZL2 were developed to encapsulate SPIO nanocrystals and form shell-crosslinked magnetic micelles with enhanced colloidal stability and prolonged circulation time in the bloodstream. PCPZL2 was observed to be the most potent carrier for SPIO loading, and SPIO nanoclusters with small interparticle spacing and an average diameter of 101.7 ± 17.2 nm were formed in the core of SPIO-loaded PCPZL2 micelles. The r_2_ value of the SPIO-loaded PCPZL2 micelles reached 720.1 mM^−1^ s^−1^, which exceeded those of Resovist^®^ and Feridex^®^ by approximately 5.5 and 6.7 times, respectively. In addition, SPIO-loaded PCPZL2 micelles were effective in vivo for liver MRI by significantly amplifying the relative signal difference between tumor and normal liver tissues, making it easier to detect and observe lesions. Our results revealed that PCPZL2 is a promising SPIO delivery system and that SPIO-loaded PCPZL2 micelles should be thoroughly investigated and used as MRI probes for in vivo diagnostics or therapeutic applications.

## Figures and Tables

**Table 1:** Compositions and molecular weights of various copolymers.

Polymer	PEG: PZL in the Copolymer			Molecular Weight	
	Calculated Molar Ratio ^1^	Measured Molar Ratio ^2^	Measured Mass Ratio ^2^	Mn (kDa)	PDI
PZL1	-	-	-	4.23	1.17
PZL2	-	-	-	6.52	1.22
PCPZL1	1:1	1.1:1	1.1:1	12.57	1.47
PCPZL2	2:1	1.9:1	1.1:1	26.99	1.40

^1^ Calculated using the feed molar ratio of SCM-PEG-SCM to PZL; ^2^ Determined from ^1^H NMR.

## Data Availability

The data are available upon request.
